# Adamantylidene-substituted alkylphosphocholine TCAN26 is more active against *Sporothrix schenckii* than miltefosine

**DOI:** 10.1590/0074-02760160088

**Published:** 2016-07-11

**Authors:** Luana Pereira Borba-Santos, Kelly Ishida, Theodora Calogeropoulou, Wanderley de Souza, Sonia Rozental

**Affiliations:** 1 Universidade Federal do Rio de Janeiro, Centro de Ciências da Saúde, Instituto de Biofísica Carlos Chagas Filho, Laboratório de Biologia Celular de Fungos, Rio de Janeiro, RJ, Brasil; 2 Universidade de São Paulo, Instituto de Ciências Biomédicas, São Paulo, SP, Brasil; 3 National Hellenic Research Foundation, Institute of Biology, Medicinal Chemistry and Biotechnology, Athens, Greece; 4 Universidade Federal do Rio de Janeiro, Instituto Nacional de Ciência e Tecnologia de Biologia Estrutural e Bioimagem, Rio de Janeiro, RJ, Brasil

**Keywords:** Sporothrix schenckii, antifungal, miltefosine analogue

## Abstract

Sporotrichosis is the most frequent subcutaneous mycosis in the world and its increasing incidence has led to the search for new therapeutic options for its treatment. In this study, we demonstrated that three structural analogues of miltefosine (TCAN26, TC19, and TC70) showed inhibitory activity against *Sporothrix schenckii sensu stricto* and that TCAN26 was more active in vitro than miltefosine against several isolates. Scanning electron microscopy showed that *S. schenckii* exposure to TCAN26 resulted in cells that were slightly more elongated than untreated cells. Transmission electron microscopy showed that TCAN26 treatment induced loss of the regular cytoplasmic electron-density and altered the cell envelope (disruption of the cell membrane and cell wall, and increased cell wall thickness). Additionally, TCAN26 concentrations required to kill *S. schenckii* cells were lower than concentrations that were cytotoxic in mammalian cells, and TCAN26 was more selective than miltefosine. Thus, the adamantylidene-substituted alkylphosphocholine TCAN26 is a promising molecule for the development of novel antifungal compounds, although further investigations are required to elucidate the mode of action of TCAN26 in *S. schenckii* cells.

Sporotrichosis has emerged as the most frequent subcutaneous mycosis in the world (Chakrabarti et al. 2015), and has become a major public health problem in Brazil (Freitas et al. 2014). It is caused by *Sporothrix* spp., of which *Sporothrix schenckii sensu stricto* is one of the most virulent species that is frequently isolated from human cases globally (Zhang et al. 2015). Despite being a disease with an increasing incidence worldwide (Chakrabarti et al. 2015), there are few therapeutic options available for sporotrichosis treatment. While itraconazole is the ‘gold standard’ treatment for the cutaneous and lymphocutaneous forms, amphotericin B is the first-line drug of choice for disseminated forms of the disease (Kauffman et al. 2007). However, itraconazole therapy involves prolonged treatment and is expensive, and amphotericin B therapy induces several side effects. Therefore, search for new therapeutic options for sporotrichosis treatment is necessary.

Recently, the antifungal activity of miltefosine, an alkylphospholipid analogue, was described against *S. schenckii* complex species (Brilhante et al. 2014, Borba-Santos et al. 2015). Miltefosine’s cytotoxicity, although considerable, is comparable to that of amphotericin B (Borba-Santos et al. 2015). Efforts to reduce miltefosine cytotoxicity have led to the synthesis of less toxic and more active alkylphospholipid analogues based on miltefosine’s structure (Avlonitis et al. 2003, Calogeropoulou et al. 2008, Papanastasiou et al. 2010). The purpose of this study was to verify the inhibitory activity of eight structural analogues of miltefosine against *S. schenckii sensu stricto*, and evaluate the antifungal activity and selectivity of the most active compound, in comparison with those of miltefosine.

Therefore, we used 12 human isolates of *S. schenckii sensu stricto* (described from here only as *S. schenckii*) to evaluate the anti-*Sporothrix* activity of eight structural analogues of miltefosine (Fig. 1): four reference strains (ATCC MYA 4820, ATCC MYA 4821, ATCC 32286, ATCC 16345) and eight clinical isolates (SB02, BH1, Ss 03, Ss 22, Ss 59, Ss 73, Ss 116, Ss 144) (Borba-Santos et al. 2016).

**Fig. 1:**
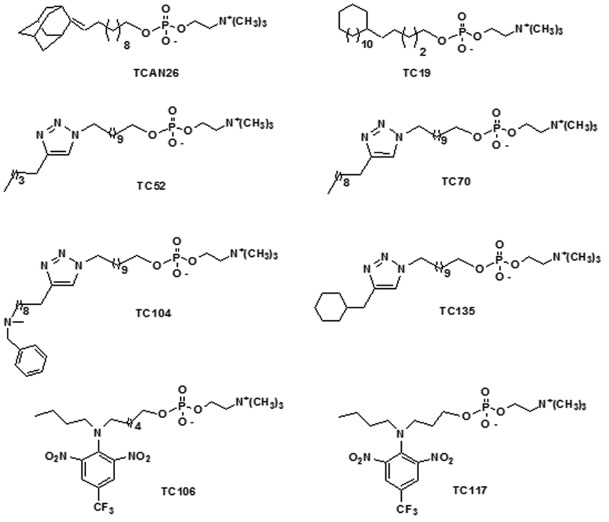
: structures of structural analogues of miltefosine tested in the present study.

The isolates were maintained in potato dextrose agar (PDA; Difco, Detroit, USA) at 4ºC. Before the experiments, the isolates were cultivated in PDA medium at 35ºC for seven days, to obtain the filamentous form, or in brain heart infusion (BHI; Difco, Detroit, USA) broth supplemented with 2% glucose at 36ºC with orbital agitation (150 rpm) to obtain the yeast phase. The structural analogues of miltefosine were diluted in DMSO:ethanol (1:1) to obtain stock solutions of 50 mM. TCAN26 and TC19 were synthesized as previously described (Avlonitis et al. 2003, Calogeropoulou et al. 2008), while the synthesis of other compounds will be reported hereafter. Miltefosine (Cayman Chemical Company, MI, USA) was used as a control and was diluted in distilled water to obtain stock solutions of 2 mg/mL, maintained at -20ºC.

Antifungal activity of compounds was evaluated according to minimum inhibitory concentrations (MICs) and minimum fungicidal concentrations (MFCs). MIC values were obtained for *S. schenckii* using a broth microdilution method adapted from M27-A3 (yeast) or M38-A2 (filamentous form) documents of Clinical and Laboratory Standards Institute (CLSI 2008a, b), with the following minor modifications: (i) RPMI 1640 medium was supplemented with 2% glucose and (ii) incubation time was extended to five days. Yeast (0.5-2.5 x 10^3^ CFU/mL) or conidial suspension (0.5-2.5 x 10^4^ CFU/mL) was treated with miltefosine analogues or miltefosine for five days at 35ºC in a dark, humid chamber with 5% CO_2_. MIC was defined as the lowest concentration that prevents visible fungal growth in an inverted optical microscope. MFC values were obtained by plating, in drug-free PDA, 10 µL aliquots collected for the inhibitory concentrations (at the end of the five-day incubation period), and incubating the aliquots at 35ºC for seven days. MFC was defined as the lowest drug concentration that failed to yield fungal growth. When MFC ≤ 4 x MIC, the agent is considered to be fungicidal, whereas MFC > 4 x MIC indicates fungistatic activity (Pfaller et al. 2004). Statistical analysis was performed using Graph Pad Prism 5.0 (Graph Pad Software Inc., USA), wherein Mann Whitney’s test was applied to analyse differences in susceptibility between the filamentous form and yeast, and correlations between MIC values were analysed by linear regression test. Statistically significance was set at p < 0.05 and positive correlation was considered if r > 0.5 and p < 0.05.

*S. schenckii* ATCC MYA 4821 - strain of the Genome Project (Teixeira et al. 2014) - was used to perform the electron microscopy analyses. Yeasts were treated with a sub-inhibitory concentration (1/4 MIC) of the most active miltefosine analogue, for 24 h at 35ºC. Untreated and treated cells were washed three times in PBS and fixed in a solution of 2.5% glutaraldehyde and 4% formaldehyde in 0.1 M cacodylate buffer (pH 7.2) for 24 h at 4ºC. For scanning electron microscopy (SEM) visualisation, cells were plated on to poly-L-lysine-covered glass coverslips and post-fixed in 1% osmium tetroxide in 0.1 M cacodylate buffer containing 1.25% potassium ferrocyanide and 5 mM CaCl_2_ for 30 min. They were then washed and dehydrated in increasing ethanol concentrations ranging from standard to ultra-dry ethanol, critical-point-dried in CO_2_ and coated with gold. The images were obtained using FEI Quanta 250 scanning electron microscope (FEI, Netherland). Feret diameters (average of distances between two parallel lines tangential to the particle projection) of 50 cells exhibiting yeast morphology were measured using Image J software (NHI, USA) and cell sizes were estimated according these values. Aspect ratio (ratio between maximum and minimum Feret diameters) was calculated to reflect the shape of cells. Values closer to one indicate a globose/oval morphology. Values much higher or smaller than one indicate an elongated cell morphology. Differences in cell size were analysed using Mann Whitney’s test and p < 0.05 was considered statistically significant (Graph Pad Software Inc., USA). For transmission electron microscopy (TEM), cells were post-fixed in 1% osmium tetroxide in 0.1 M cacodylate buffer, containing 1.25% potassium ferrocyanide and 5 mM CaCl_2_, for 2 h at 4ºC, dehydrated in ethanol, and embedded in Spurr resin. Ultrathin sections were stained with uranyl acetate and lead citrate and observed under Zeiss 900 transmission electron microscope (Zeiss, Germany). Thickness of cell walls was measured using Image J software in 50 cells per sample. Differences in cell wall thickness were analysed by Mann Whitney’s test, with p < 0.05 considered statistically significant (Graph Pad Software Inc., USA).

To determine the selectivity of the most active miltefosine analogue toward *S. schenckii*, cytotoxicity assays were performed on mammalian cells [monkey epithelial cells from kidney (LLC-MK2)] and human erythrocytes, using concentrations of 1-100 µg/mL as described previously (Borba-Santos et al. 2016), and the results were compared with those obtained for miltefosine. CC_50_ (TCAN26 concentration that impaired viability in 50% of LLC-MK2 cells) and HA_50_ (TCAN26 concentration that caused lysis in 50% of erythrocytes) values were determined and the antifungal drugs’ selectivity indexes (SI) were obtained using the following equation: SI = CC_50_ or HA_50_/MIC medians.

Among all miltefosine analogues tested against the reference strain *S. schenckii* ATCC MYA 4821 (Fig. 1), only three (TCAN26, TC19 and TC70) showed considerable inhibitory activity (MIC < 16 µg/mL). The adamantylidene-substituted alkylphosphocholine, TCAN26, was found to be the most active compound against both yeast (MIC = 0.5 µg/mL) and filamentous forms (MIC = 1.0 µg/mL) (Table I). Therefore, TCAN26 was chosen to expand the susceptibility analyses to other isolates (including three reference strains and eight clinical isolates) (Table II, Supplementary Table).

**TABLE I:** Minimum inhibitory concentration (MIC) of eight structural analogues of miltefosine, compared to miltefosine, against *Sporothrix schenckii* ATCC MYA 4821 isolate in filamentous and yeast forms (µg/mL)

Compounds		MIC	

		Filamentous	yeast
Cycloalkylphospholipids	Miltefosine	1	1
	TCAN26	1	0.5
	TC19	2	2
Alkyltriazolylphospholipids	TC52	>16	> 16
	TC70	2	2
	TC 104	>16	> 16
	TC 135	>16	> 16
Alkylphospholipid-dinitroaniline hybrids	TC106	>16	> 16
	TC 117	>16	> 16

**TABLE II:** Antifungal activity of TCAN26, compared to that of miltefosine, against *Sporothrix schenckii* isolates in filamentous and yeast forms (µg/mL)

		MIC range^a^	MIC median^b^	MIC 50^c^	MIC 90^d^	MFC range^e^	MFC median^f^	MFC 50^g^	MFC 90^h^
Miltefosine	Filamentous form	0.5-2	2	2	2	1-16	2	2	4
	Yeast	0.5-2	2	2	2	1-4	2	2	4
TCAN26	Filamentous form	0.5-1	1	1	1	0.5-8	1	1	4
	Yeast	0.25-2	1	1	2	0.5-4	2	1	2

*a*: range of minimum inhibitory concentration (MIC) values (lowest drug concentrations that inhibited relative to untreated controls) for the different fungal strains tested; *b*: medians of MIC values; *c*: concentration that inhibited growth in 50% of isolates; *d*: concentration that inhibited growth in 90% of isolates; *e*: range of minimum fungicidal concentration (MFC) values (defined as the lowest drug concentrations that produced no fungal growth) for the fungal isolates tested; *f*: medians of MFC values; *g*: concentration that produced no fungal growth in 50% of isolates; *h*: concentration that produced no fungal growth in 90% of isolates.

According to MIC values, TCAN26 was more active than miltefosine (MIC median equal to 1 and 2 μg/mL, respectively) against *S. schenckii* yeasts (p = 0.0187) and the filamentous form (p = 0.003). MIC values for TCAN26 ranged from 0.25 to 2 µg/mL for yeasts and from 0.5 to 1 µg/mL for filamentous forms, whereas MIC values for miltefosine ranged from 0.5 to 2 µg/mL for yeasts and filamentous forms (Table II). No statistically significant differences were observed between MIC values of TCAN26 for filamentous form and yeasts (p = 0.8408); similar findings were observed for miltefosine (p = 0.5732). Positive correlations were obtained for MIC values in filamentous and yeast forms for TCAN26 (r = 0.7588 and p = 0.0026) and miltefosine (r = 0.6721 and p = 0.0119). These data indicate that both forms were equally sensitive to TCAN26 and miltefosine. MFC values for TCAN26 ranged from 0.5 to 4 µg/mL for yeasts and from 0.5 to 8 µg/mL for filamentous forms, whereas MFC values for miltefosine ranged from 1 to 4 µg/mL for yeasts and from 1 to 16 µg/mL for filamentous forms. Additionally, MFC values suggested that TCAN26, similar to miltefosine, showed fungicidal activity (MFC ≤ 4 x MIC) (Table II, Supplementary Table).

To assess ultrastructural alterations after exposure to TCAN26, yeasts of the reference isolate ATCC MYA 4821 were treated with 0.125 µg/mL TCAN26 (1/4 MIC), for 24 h, and processed by SEM and TEM. SEM images revealed that the control sample exhibited yeast cells with an elongated morphology and some hyphae cells (white arrow in Fig. 2A), and TCAN26 exposure did not induce pronounced superficial changes in cells (Fig. 2C). Feret diameter analyses indicated no alterations in yeast size after TCAN26 treatment (p > 0.05) (hyphae and undetermined cells were disregarded for this analysis), but yeasts treated with TCAN26 were slightly more elongated than control yeasts (aspect ratio mean equal to 3.33 ± 0.16 and 3.13 ± 0.14, respectively). TEM images showed that control cells exhibited homogenous and electron-dense cytoplasm containing nucleus (N), vacuoles (v) and several mitochondria (m), and surrounded by cell membrane and cell wall (Fig. 2B and inset). TCAN26 exposure reduced cytoplasmic electron-density (Fig. 2D) and induced disruption of the cell membrane and cell wall (arrowheads in Fig. 2D). The cell wall of TCAN26-treated cells (mean of 163.7 nm) was thicker than that of control cells (mean of 142.2 nm) (p = 0.0006).

**Fig. 2:**
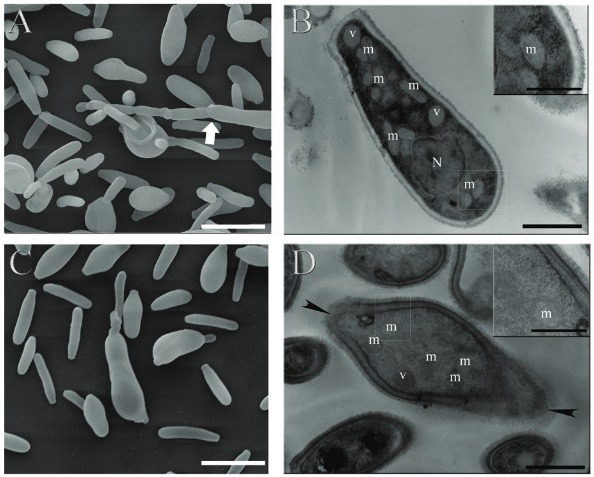
: ultrastructural alterations of *Sporothrix schenckii* ATCC MYA 4821 on exposure to TCAN26, evaluated by scanning electron microscopy (A, C) and transmission electron microscopy (B, D). Control cells (untreated) exhibit yeasts and some hyphae cells (Fig. 2A, white arrow) while samples treated with 0.125 µg/mL TCAN26 for 24 h show only yeasts (Fig. 2C). Control cells exhibit homogenous and electron-dense cytoplasm containing nucleus (N), vacuoles (v), and several mitochondria (m), and are surrounded by the cell membrane and cell wall (Fig 2B and inset). TCAN26 exposure-induced reduction of cytoplasmic electron-density (Fig. 2D), disruption of cell membrane and cell wall (arrowheads in Fig. 2D) and increase in the cell wall thickness (inset in Fig. 2D) (Bars: A, C: 5 µm; B, D: 1 µm; insets in B and D: 0.5 µm).

Selectivity of TCAN26 towards *S. schenckii* was evaluated according to CC_50_ and HA_50_ values determined in LLC-MK2 cells and human erythrocytes, respectively. TCAN26 was 23 times more selective for fungus than for LLC-MK2 cells and 52 times more selective for fungus than for erythrocytes. TCAN26 was also more selective for fungus and less cytotoxic to mammalian cells than miltefosine was (Table III).

**TABLE III:** Selectivity of TCAN26, compared to that of miltefosine, for *Sporothrix schenckii*

		Miltefosine	TCAN26
MIC medians^a^	Filamentous form	2	1
	Yeast	2	1
LLC-MK2 cytotoxicity	CC_50_^b^ (µg/mL)	5^e^	23
	SI^c^ filamentous form	2.5	23
	SI^c^ yeast	2.5	23
Erythrocytes lysis	HA_50_^d^ (µg/mL)	18	52
	SI^c^ filamentous form	9	52
	SI^c^ yeast	9	52

*a*: medians of minimum inhibitory concentration values (MIC); *b*: 50% cytotoxic concentration; *c*: selective index (SI) = CC_50_ or HA_50_/MIC medians; *d*: 50% haemolytic concentration; *e*: CC_50_ value reported in Borba-Santos et al. (2015).

Currently, sporotrichosis is the major subcutaneous fungal infection (Chakrabarti et al. 2015). The availability of only a few therapeutic options warrants a search for more effective antifungal agents. Here, we demonstrated that three structural analogues of miltefosine (TCAN26, TC19 and TC70) inhibited fungal growth and TCAN26 was more selective than miltefosine against *S. schenckii*.

The antimicrobial activity of TCAN26 was previously reported in *Leishmania* spp. with its inhibitory activity ranging from 1 to 20 µg/mL, depending on the species (Calogeropoulou et al. 2008). TCAN26 reportedly also showed inhibitory activity against *Candida albicans* yeasts (MIC equal to 4 µg/mL) and biofilms (Vila et al. 2013). Interestingly, our results showed that TCAN26 was more active against *S. schenckii* than it was against previously reported microorganisms, impairing fungal growth with concentrations ranging from 0.25 to 2 µg/mL. In addition, TCAN26 showed a fungicidal activity profile against *S. schenckii* (Table II, Supplementary Table).

In this work, we also demonstrated that TCAN26 was more selective for fungal cells (Table III) than for mammalian cells (23 times more selective than for LLC-MK2 cells and 52 times more selective than for human erythrocytes). Considering fungal MFC, the TCAN26 concentrations required to kill *S. schenckii* cells (≤ 8 µg/mL) were lower than the concentrations cytotoxic to LLC-MK2 cells (CC_50_ = 23 µg/mL) and lower than the concentrations that caused lysis in erythrocytes (HA_50_ = 52 µg/mL).

Being an alkylphospholipid analogue, TCAN26 probably alters cellular lipid homeostasis in a manner similar to that observed with miltefosine (Marco et al. 2014). *S. schenckii* exposure to TCAN26 resulted in cells that were slightly more elongated than control cells (untreated) (Fig. 2A-C), and this treatment induced loss of the regular cytoplasmic electron-density and altered the cell envelope (disruption of the cell membrane and cell wall, and increased cell wall thickness) (Fig. 2D). *C. albicans* treated with TCAN26 also showed cell membrane alterations and increase in cell wall thickness (Vila et al. 2013).

In conclusion, this study suggests that the adamantylidene-substituted alkylphosphocholine TCAN26 is a promising molecule for the development of new antifungal compounds, although further investigations need to be conducted to elucidate the mode of action of TCAN26 in *S. schenckii* cells.

## SUPPLEMENTARY TABLE

**Table:** Antifungal activity of TCAN26 and miltefosine against *Sporothrix schenckii* isolates in filamentous and yeast forms (µg/mL)

Strains	Source	Miltefosine				TCAN26			
	
		Filamentous		Yeast		Filamentous		Yeast	
			
		MIC	MFC	MIC	MFC	MIC	MFC	MIC	MFC
ATCC MYA 4821	human isolate - USA	1	2	1	1	1	2	0.5	4
ATCC MYA 4820	human isolate - Brazil	2	2	2	2	1	2	2	2
ATCC 32286	human isolate - Brazil	1	1	0.5	2	0.5	0.5	1	1
ATCC 16345	human isolate - USA	0.5	1	1	2	1	1	0.25	0.5
SB02	human isolate - Brazil	2	2	1	2	1	4	0.5	1
BH1	human isolate - Brazil	1	2	1	2	0.5	0,5	0.5	1
Ss 03	human isolate - Brazil	2	2	2	2	1	8	1	2
Ss 22	human isolate - Brazil	2	2	2	2	1	2	2	2
Ss 59	human isolate - Brazil	2	2	2	2	1	1	1	1
Ss 73	human isolate - Brazil	2	16	2	4	1	1	2	2
Ss 116	human isolate - Brazil	2	2	2	2	1	1	1	1
Ss 144	human isolate - Brazil	2	4	2	2	1	1	1	2

MIC: minimum inhibitory concentration; MFC: minimum fungicidal concentration.
